# Supplementary figures and images for: Fluorescence-based index from the Sofia Streptococcus pneumoniae fluorescent immunoassay: a prognostic tool for pneumococcal community-acquired pneumonia
Source: Microbiol Spectr. 2026 Mar 6;14(4):e03304-25. doi: 10.1128/spectrum.03304-25 (PMC13055352; doi:10.1128/spectrum.03304-25)

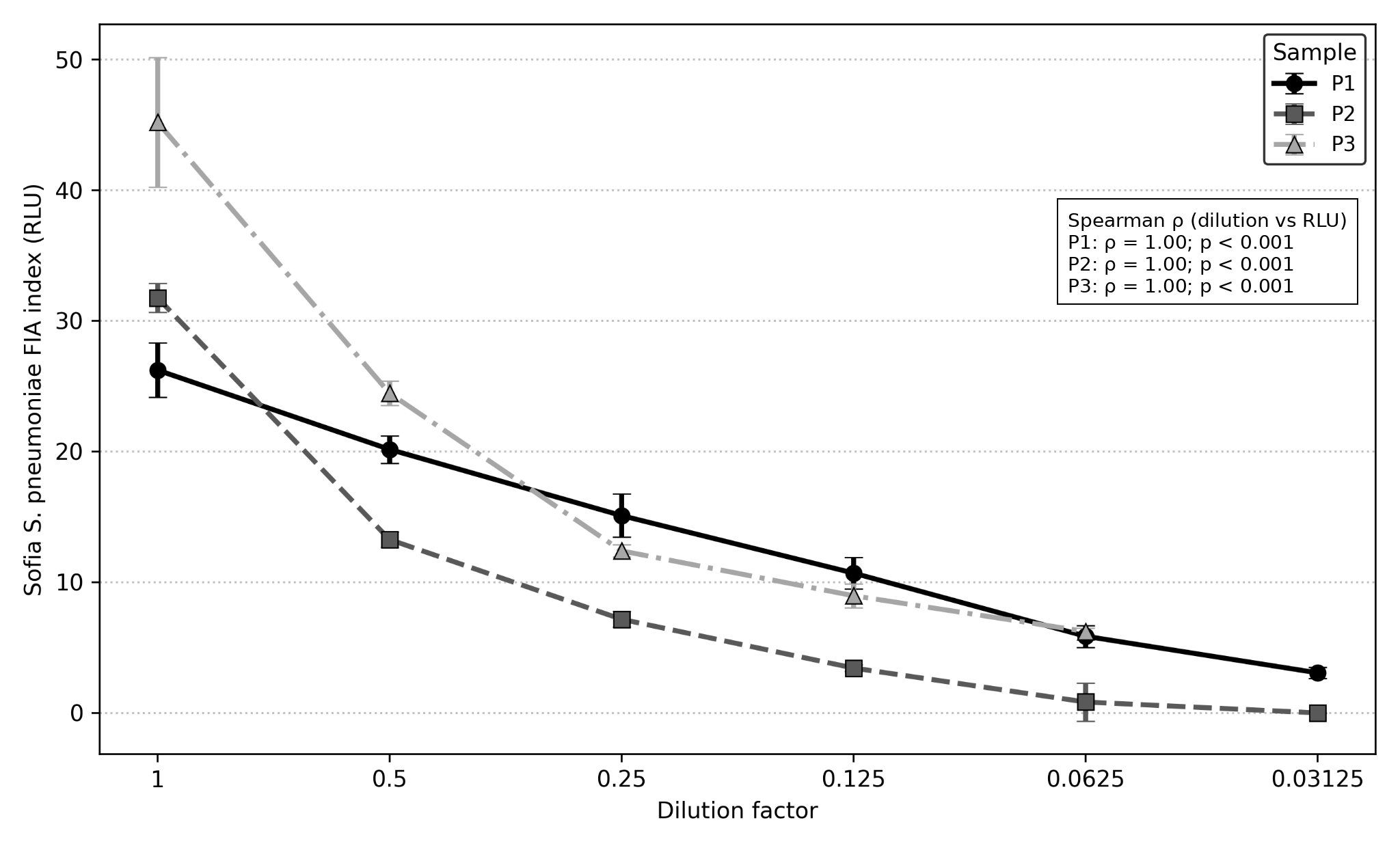

Supplement: Figure S1 — Serial dilution response of the Sofia S. pneumoniae FIA index. Three urine samples were tested in triplicate across a 1:2 serial dilution series. Points represent mean RLU values and error bars indicate ± Standard deviation. The x-axis shows dilution factors (most concentrated at 1, followed by sequential dilutions). [file spectrum.03304-25-s0001.tif]
