# Supplementary material for: Fluorescence-based index from the Sofia Streptococcus pneumoniae fluorescent immunoassay: a prognostic tool for pneumococcal community-acquired pneumonia
Source: Microbiol Spectr. 2026 Mar 6;14(4):e03304-25. doi: 10.1128/spectrum.03304-25 (PMC13055352; doi:10.1128/spectrum.03304-25)
Supplement: Supplemental legend — Legend for Figure S1. [file spectrum.03304-25-s0002.docx]

**Supplementary Figure S1. Serial dilution response of the Sofia S. pneumoniae FIA index.** Three urine samples were tested in triplicate across a 1:2 serial dilution series. Points represent mean RLU values and error bars indicate ± Standard deviation. The x-axis shows dilution factors (most concentrated at 1, followed by sequential dilutions).
